# Adolescent and parental decision-making for the MenACWY vaccination: influential predictors and parental-adolescent differences among households in the Netherlands

**DOI:** 10.1186/s12889-023-15872-9

**Published:** 2023-05-25

**Authors:** C. Oostdijk, J. A. Ferreira, W. L. M. Ruijs, L. Mollema, K. Van Zoonen

**Affiliations:** 1 Centre for Infectious Disease Control, National Institute for Public Health and the Environment (RIVM), PO Box 1, 3720 BA Bilthoven, The Netherlands; 2 Department of Statistics, Informatics and Mathematical Modeling, National Institute for Public Health and the Environment (RIVM), PO box 1, Bilthoven, 3720 BA the Netherlands

**Keywords:** Adolescent vaccination, Decision-making, Vaccination catch-up campaign, Vaccination acceptance, Meningococcal disease, MenACWY

## Abstract

**Background:**

Between 2015 and 2018 The Netherlands experienced increases of invasive meningococcal disease (IMD) serogroup W (MenW). Therefore in 2018 the MenACWY vaccination was introduced in the National Immunisation Programme (NIP) and a catch-up campaign was initiated targeting adolescents.

This study aimed to gain insight into what factors played a role in the decision-making process regarding the MenACWY vaccination. The focus was on the differences in the decision-making of parents and adolescents in order to assess what factors influence the decisions made.

**Methods:**

An online questionnaire was offered to adolescents and one of their parents. We used random forest analyses to determine which factors best predict the outcome of the MenACWY vaccination decision. We carried out ROC (receiver-operator characteristics) analyses to confirm the predictive value of the variables.

**Results:**

Among parents several factors stand out, centring on the process of the decision, their attitude about the MenACWY vaccination, trust in the vaccination, and ideas of important people around them. Among adolescents the three stand-out predictors are the ideas of important people around them, the process of the decision and trust in the vaccination.

Parents have prominent influence in the decision-making, while the adolescent’s influence in the household decision-making is more limited. Adolescents tend to be less engaged and spend less time thinking about the decision compared to parents. Opinions of parents and adolescents from the same households concerning the factors that are influential do not differ a lot in the final decision-making.

**Conclusions:**

Information about MenACWY vaccination might be mainly addressed to the parents of the adolescents and whereby the dialogue about MenACWY vaccination between parents and adolescents will be stimulated. With regard to the predictor trust in vaccination, raising the frequency of use of certain sources, especially those deemed very reliable among households such as conversations with a GP or the provider of the vaccination (GGD/JGZ), might prove a useful strategy to solidify vaccination uptake numbers.

**Supplementary Information:**

The online version contains supplementary material available at 10.1186/s12889-023-15872-9.

## Background

A number of countries have dealt with outbreaks of invasive meningococcal disease (IMD) in the past decades, with serogroup W (MenW) cases linked to high fatality rates [1–4]. Between 2015 and 2018 the Netherlands also experienced increases of IMD (MenW) with incidence rates having risen from 0.02 cases/100,000 between 2010–2014 up to 0.5 cases/100,000 in 2017 [5, 6]. The highest incidence rates at the time were found among those below five years old and among 15 – 24 year olds [5, 7]. Due to these developments, the MenACWY vaccine was introduced into the Dutch National Immunisation Programme (NIP) in 2018. The MenACWY vaccination replaced the MenC vaccination already in use (since 2002) to vaccinate 14-month-olds. The MenACWY vaccination was simultaneously introduced into the NIP for adolescents turning 14 years old. This was decided because adolescents were both a vulnerable group themselves and considered an important carrier of meningococci bacteria, so the introduced policy intended to indirectly also protect other vulnerable groups at risk [6, 8, 9].

The MenACWY vaccination was introduced among adolescents through a targeted catch-up campaign in late 2018 for those born between May 2004 and December 2004. This was expanded to include all adolescents born between 2001 and 2005 the following year [6]. Around the time of the catch-up campaign adolescents had not been targeted much within the Dutch NIP, with the only other vaccination offered around that age being the HPV vaccination for girls aged 12 years old.

Among eligible adolescents targeted in the catch-up campaign, the eventual uptake of the MenACWY vaccination was 86%. However, regional differences and varying uptake numbers among different population characteristics indicate the benefits of getting more insight into the decision-making process [6, 10, 11].

Parental decision-making concerning childhood vaccinations has been widely researched. Many studies look at which factors influence intentions and what reasons people have for vaccination decisions, demonstrating that oftentimes people make decisions based on their attitudes and beliefs concerning vaccination in general [12–17]. Studies concerning adolescent vaccinations have covered either parental or adolescent perspectives separately or incorporated both into a comparison of the two highlighting differences between them (e.g., in attitude, beliefs or intention to vaccinate) [1, 17–27].

The goal of our study is to gain insight into which factors contribute to choices made by parents and adolescents concerning the MenACWY vaccination in the Netherlands using random forest [28]. By only including households that had gone through the decision-making as part of the catch-up campaign, we are able to evaluate actual behaviour instead of intention. This allows us to look into what actually happened during the decision-making.

Furthermore, we wanted to identify and understand possible differences between the ideas of adolescents and those of their parent (within the same household) regarding the MenACWY vaccination decision-making. Additionally, we looked at how respondents make such a decision (e.g., moments of information-seeking, inter-household dynamics, doubts, barriers, household-member involvement) and categorical questions pertaining to these themes were included in the survey.

Insight into the main factors that influence the ideas of parents and adolescents about vaccination, combined with insight into agreements and disagreements between them regarding those factors, help us to understand the decision-making process involved in MenACWY vaccination in the Netherlands. This can be of use in the planning and implementation of future vaccination (catch-up) campaigns targeting adolescents by better catering to those involved.

## Methods

This study used a quantitative approach through an online questionnaire offered to adolescents born in 2004 and 2005 (at the time, aged between 13 and 15 years) who were invited for the MenACWY vaccination catch-up campaign in either 2018 or 2019. A questionnaire was also offered to one of their parents. We invited adolescents and parents from the same households and encouraged both to participate separately. However, either could also participate solo.

All participants were invited through Praeventis, the national vaccination register of the National Institute for Public Health and the Environment (RIVM). Each household received an invitation letter and an accompanying flyer about the study via post. Those who choose to participate gave written informed consent at the start of the online questionnaire. Adolescents were specifically prompted to also get parental consent before commencing. Both in the invitation and before starting the questionnaire, participants were informed about their privacy and the steps taken to assure confidentiality. They were explicitly informed of their right to withdraw participation, without having to provide justification for choosing to do so. All data provided were anonymised and researchers are unable to link answers to any person or address.

Each invitation was labelled with a randomised unique number. People from the same household were asked to enter this number within their respective questionnaires so as to allow for the identification of parent-adolescent dyads.

As per policy of the Praeventis database administrator at the time of the research, no reminders to participate were sent to those invited.

The research was exempt from ethical review as decided by the Medical Ethics Review Committee (METC) MedNec based in Utrecht, The Netherlands (reference number 19–110/C).

### Participants

Adolescents and parents from cohort 2004 were invited to participate from 26 June 2019 to 24 July 2019. Adolescents and parents from cohort 2005 were invited to participate from 3 October 2019 to 31 October 2019.

**Fig. 1:**
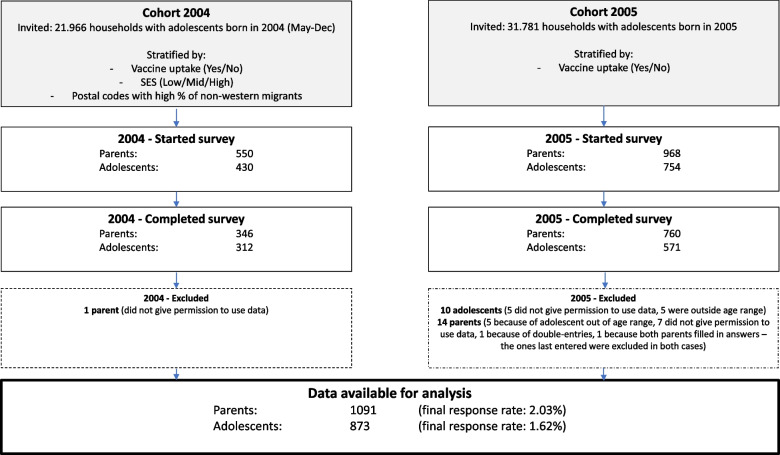
Flow chart with an overview of invited and participated parents (*N* = 1,091) and adolescents (*N* = 873) (born in 2004 or 2005) within households

To calculate the necessary invites to be sent out, we adhered to a response rate of 9% of people willing to participate in the research among those that had obtained the MenACWY vaccination. For those who had not obtained the MenACWY vaccination, those in postal code areas with lower socioeconomic status (SES) averages and those in postal code areas with high percentages of people with a non-western migration background, we adhered to a lower response rate of 4% to participate in the study, and thus we oversampled among these groups. For the adolescents born in 2004 a random sample was taken from all postal codes in the Netherlands stratified by vaccine uptake, SES and migration background. All adolescents born in 2005 from three largest cities in the Netherlands (Amsterdam, Utrecht and The Hague) were invited and an additional random sample was taken from the rest of the Netherlands for those who were vaccinated against MenACWY and those who were not. For cohort 2005 no extra stratification was done by SES or migration background. See also Fig. 1 with an overview of invited and participated parents and adolescents within households.

We first recruited participants among cohort 2004 before expanding our study to include respondents from cohort 2005. We recruited among specifically these cohorts as they were the first to be invited (cohort 2004) and best resembled households that might be targeted by other vaccination campaigns concerning adolescents (cohort 2004 and 2005).

### Questionnaire

**Fig. 2:**
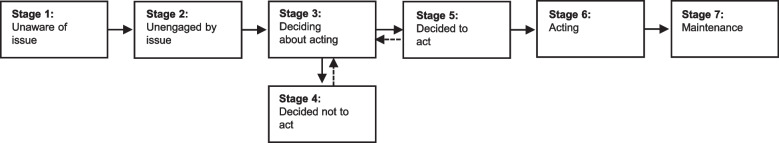
The Precaution Adoption Process (PAP) Model describing how people make a decision about and act upon health-protective behaviours such as vaccination

The Precaution Adoption Process (PAP) model formed the theoretical framework of this study [29, 30]. The PAP model (see Fig. 2) theorises how people make a decision about and act upon health-protective behaviours. It frames behavioural change as a process and conceives that people must become aware and become engaged before thinking about the possibility, then deciding whether to adopt a behaviour or not and finally to act upon that decision [29, 30].

The questionnaire was based on previous literature on factors influencing vaccination decision-making. Included variables covered attitude [31, 32], deliberation [33], risk perception [34], trust [35], beliefs [27, 36], norms [27, 37] and decisional conflict [38]. These questions were posed using 5-point Likert scale answer options.

Additionally, actions of parents and adolescents taken before and during the various stages of the decision-making process (e.g., reading the folder, searching for information, discussing the topic, being involved in the decision) were included in the questionnaire. These questions were posed as categorical questions with predefined answer categories, but respondents had the option to add their own answers with some. Of these, answers deemed to fit in one of the categories were coded as such, but most were very diverse and left as other.

Both parents and adolescents were asked about similar constructs in their questionnaires, but sometimes worded slightly different to clarify to whom the question pertained and to accommodate adolescent language levels.

The questionnaire was pretested among members of the target population and subsequently revised. An additional revision occurred in the questionnaire for parents of the 2005 cohort. In this case, the questionnaire was shortened based on feedback from parents from cohort 2004.

### Analyses

To determine which factors best predict the outcome of the MenACWY vaccination decision, random forest (RF) analyses were performed. Of the many statistical predictors available, RF is one of the most accurate and one that is likely to approximate the data-generating mechanism reasonably well and hence to provide a reasonably accurate picture of which variables are most predictive of the outcome. RFs are especially useful in our case, where several of the predictor variables are interconnected and their joint workings provides insight into people’s decision-making. We assessed which factors best predict the outcome variable (i.e., MenACWY vaccination uptake). All respondents (1,091 parents and 873 adolescents) were included in these analyses. To look at the different factors for both parents and adolescents, separate RF models were built for each group. Four variables contained some cases of missing data, due to the questions not marked as mandatory in the survey. Instead of a data imputation to append the dataset, the missing values were replaced with a ‘special value’ (i.e. a symbol that the RF analysis views as something other than a value that needs to be included in the prediction models).

The RF models produce variable importance rankings and show how much the independent variables contribute to the prediction of the outcome variable. Due to a smaller proportion of non-vaccinated individuals, we ran RF models with 1:1 and 2:1 ratios of vaccinated and non-vaccinated respondents to check whether the ranking of the stronger predictor variables was dependent on the proportion of non-vaccinated. In addition, we carried out ROC (receiver-operator characteristics) analyses to confirm the predictive value of the variables and to explore the possibility of improving the accuracy of the predictions [39].

Furthermore, we used univariate descriptive statistics to describe data and in a number of instances analysed differences between those that vaccinated and those that did not through simple logistic regression.

## Results

### Study population and decision-making process within households

Table 1 shows the total population included, with information presented separately for parents and adolescents that participated. More mothers than fathers participated in our study and most were highly educated. Among adolescents, most were born in 2005, the majority had vaccinated against MenACWY and most also adhered fully to the entire NIP. In the Netherlands, when someone is eligible for a vaccination as part of a vaccination programme, they receive an invitation in the mail. When people do not get vaccinated, a reminder gets sent out at a later date. Among all parents (*n* = 1091) 80% stated their household received only one invitation to get vaccinated. Others stated they had received more than one (14%) or were unsure about the number of invites they had received (6%).

**Table 1:** Total study population with the number of participating parents (*N* = 1,091) and adolescents (*N* = 873) and describing various characteristics

	**Parents (*n* = 1,091)**	**Adolescents (*n* = 873)**
	*n (%)*	*n (%)*
**Sex**		
Male	209 (19.2%)	405 (46.4%)
Female	882 (80.8%)	468 (53.6%)
**Adolescent vaccinated against MenACWY**		
Yes	946 (86.7%)	800 (91.6%)
No	145 (13.3%)	73 (8.4%)
**Cohort**		
2004	345 (31.6%)	313 (35.8%)
2005	746 (68.4%)	560 (64.2%)
**Migration background**		
Dutch	968 (88.7%)	817 (93.6%)
Western	55 (5.0%)	19 (2.2%)
Nonwestern	67 (6.1%)	37 (4.2%)
Missing	1 (0.1%)	-
**Education level**		
Low	55 (5.0%)	-
Middle	222 (20.4%)	-
High	814 (74.6%)	-
**Adolescent follows NIP**		
Yes, fully	949 (87.0%)	-
Yes, partly	111 (10.2%)	-
No	24 (2.2%)	-
Not sure	7 (0.6%)	-
**MenACWY vaccination an important topic**		
Completely agree	732 (67.1%)	155 (17.8%)
Agree	268 (24.6%)	397 (45.5%)
Neutral	44 (4.0%)	222 (25.4%)
Disagree	24 (2.2%)	70 (8.0%)
Completely disagree	23 (2.1%)	29 (3.3%)
**Read information folder**		
Yes	950 (87.1%)	520 (59.6%)
No	84 (7.7%)	254 (29.1%)
Do not remember	57 (5.2%)	99 (11.3%)
**Searched for additional information**		
Yes	524 (48.0%)	183 (21.0%)
No	536 (49.1%)	661 (75.7%)
Do not remember	31 (2.9%)	29 (3.3%)
**At least one parent spoke to adolescent about choice beforehand**		
Yes	848 (77.7%)	652 (74.7%)
No	191 (17.5%)	167 (19.1%)
Not sure/Do not remember/not applicable	52 (4.8%)	54 (6.2%)
**Number of invites received to get vaccinated**		
One	867 (79.5%)	593 (67.9%)
More than one	155 (14.2%)	97 (11.1%)
Do not remember	69 (6.3%)	183 (21.0%)

Among the total study population nine out of ten parents (92%) indicated they found vaccinating against meningococcal disease to be an important topic (i.e. being engaged with the topic). Fewer adolescents found the topic of the MenACWY vaccination important (63%).

According to parents, in most of their households (78%) at least one parent had a conversation about the vaccination choice with the adolescent. Just over 17% of parents indicated neither they, nor the other parent, had spoken to the adolescent about choosing for or against the MenACWY vaccination. Additionally, 67% of parents indicated to have had a conversation with the other parent about the vaccination choice.

Adolescents were also asked if and with whom they had had a conversation about the vaccination, with almost 75% of adolescents indicating they had spoken to at least one of their parent about the MenACWY decision beforehand. Here a discrepancy between the parents involved emerges, as 71% of adolescents indicated having spoken to their mother beforehand, but only 45% had spoken to their father at that stage.

Along with the invitation to get vaccinated, people received a folder with information about the disease and/or the vaccination. The majorities of both parents (87%) and adolescents (60%) stated that they had read this. Among parents 48% actively searched for additional information about the disease or vaccination prior to making a decision. Only 21% of adolescents did the same.

Simple logistic regression showed that parents not following the NIP fully for their child had a higher odds of not getting the MenACWY vaccination compared to those who do fully follow the NIP (OR = 15.35, *p* < 0.001, CI[95%] 10.16 – 23.18, *n* = 1,091).

Parents and adolescents did not necessarily come from the same households. Table 2 shows information relating only to the dyads from the same households.

**Table 2:** Study population – dyads only (*N* = 504) and describing various characteristics

	**Dyads (*n* = 504)**
	*n (%)*
**Sex (Parent)**	
Male	100 (19.8%)
Female	404 (80.2%)
**Sex (Adolescent)**	
Male	262 (52.0%)
Female	242 (48.0%)
**Adolescent vaccinated against MenACWY**	
Yes	471 (93.5%)
No	33 (6.5%)
**Cohort**	
2004	206 (40.9%)
2005	298 (59.1%)
**Migration background**	
Dutch	461 (91.5%)
Western	20 (4.0%)
Nonwestern	23 (4.5%)
**Education level (Parent)**	
Low	26 (5.1%)
Middle	102 (20.2%)
High	376 (74.6%)
**Adolescent follows NIP**	
Yes, fully	456 (90.5%)
Yes, partly	44 (8.7%)
No	2 (0.4%)
Not sure	2 (0.4%)

### Information-seeking behaviour and use of information sources

All respondents were asked through which sources they had learned anything about the disease and/or vaccination before making a decision (Table 3). This included both actively sought out sources as well as indirect encounters (e.g., the news, folder).

**Table 3:** Information sources used by parents (*N* = 1,091) and adolescents (*N* = 873) and contribution of these information sources in making a choice and the reliability of these sources

Parents				
Information sources	Used by % of parents (n) *Total n* = *1,091*	IF USED Contribution of source in making a choice (% rated very high or high)	IF USED Reliability of source (% rated very reliable)	IF USED Reliability of source (% very or somewhat)
Folder that came with invite	58.0 (633)	64.3	74.6	92.7
News	50.3 (549)	69.0	38.4	87.1
Online searches	43.9 (479)	53.4	20.5	72.0
Conversations with family/friends	27.3 (298)	59.1	28.5	73.8
RIVM website	26.2 (286)	71.7	71.3	87.1
GP, GGD, JGZ	12.8 (140)	77.9	85.0	95.7
(None used)	9.0 (98)	n/a	n/a	n/a
Other	8.5 (93)	87.1	71.0	94.6
Social Media	5.3 (58)	44.8	8.6	50.0
Adolescents				
Information sources	Used by % of adolescents (n) *Total n* = *873*	IF USED Contribution of source in making a choice (% rated very high or high)	IF USED Reliability of source (% rated very reliable only)	IF USED Reliability of source (% very or somewhat)
Conversations with parents/family	57.9 (505)	60.6	56.4	91.3
Folder that came with invite	45.4 (396)	55.3	87.3	91.9
Conversations with friends	41.1 (359)	30.6	10.9	49.9
News	39.0 (340)	45.3	45.3	84.7
Social Media	19.1 (167)	37.7	14.4	54.5
Online searches	17.9 (156)	49.4	19.2	68.0
School	6.5 (57)	43.9	36.8	80.7
None used	6.1 (53)	n/a	n/a	n/a
RIVM website	2.7 (25)	60.0	56.0	84.0
GP, GGD, JGZ	2.5 (22)	40.9	81.8	95.5
Other	1.7 (15)	40.0	33.3	66.7

The most used sources reported by parents were the folder, the news and online searches. The most used sources among adolescents were conversations with parents/family, the folder and conversations with friends.

If someone used a particular source, they were then asked how big of a contribution they believed this source had on their eventual decision. Parents rated multiple sources as making a (very) high contribution to their choice. The information sources making the most highly rated contributions were the ones deemed very reliable, and not necessarily the ones used by most people.

For adolescents, conversations with their parent(s) and the provided folder contributed to their decision and were simultaneously thought of as reliable sources. Conversations with friends stands out as often used, but not seen as very reliable among adolescents.

Social media use, although higher among adolescents, was not seen as reliable by either parents or adolescents.

### Predictors of MenACWY vaccination choice among parents and adolescents

By performing random forest (RF) analyses we looked at which of the socio-psychological factors in our survey are the most influential in retrospectively predicting vaccination choices made by parents and adolescents.

The RF model for parents has a sensitivity (i.e., probability of correctly predicting the choice of not getting the MenACWY vaccination) of 50%, a specificity (i.e., probability of correctly predicting the choice of getting the vaccination) of 99%, and a pmc (probability of misclassification) of 0.08.

**Fig. 3:**
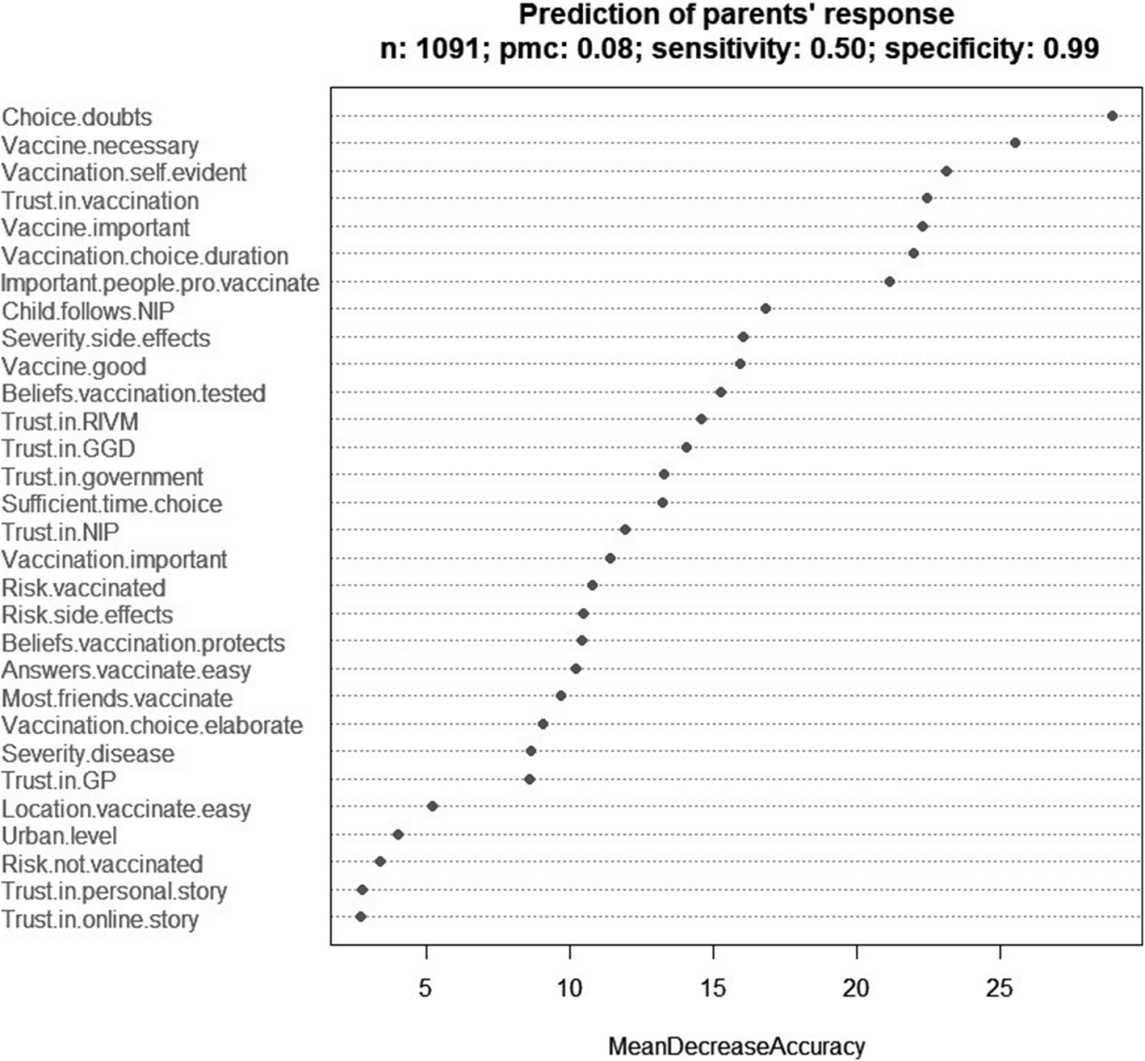
Prediction analysis: variable importance for MenACWY vaccination uptake among parents (*N* = 1,091). The Mean Decrease in Accuracy of a given predictor variable is the decrease in the proportion of correct predictions regarding MenACWY vaccination uptake that results from randomly permuting the values of that variable in the dataset

**Fig. 4:**
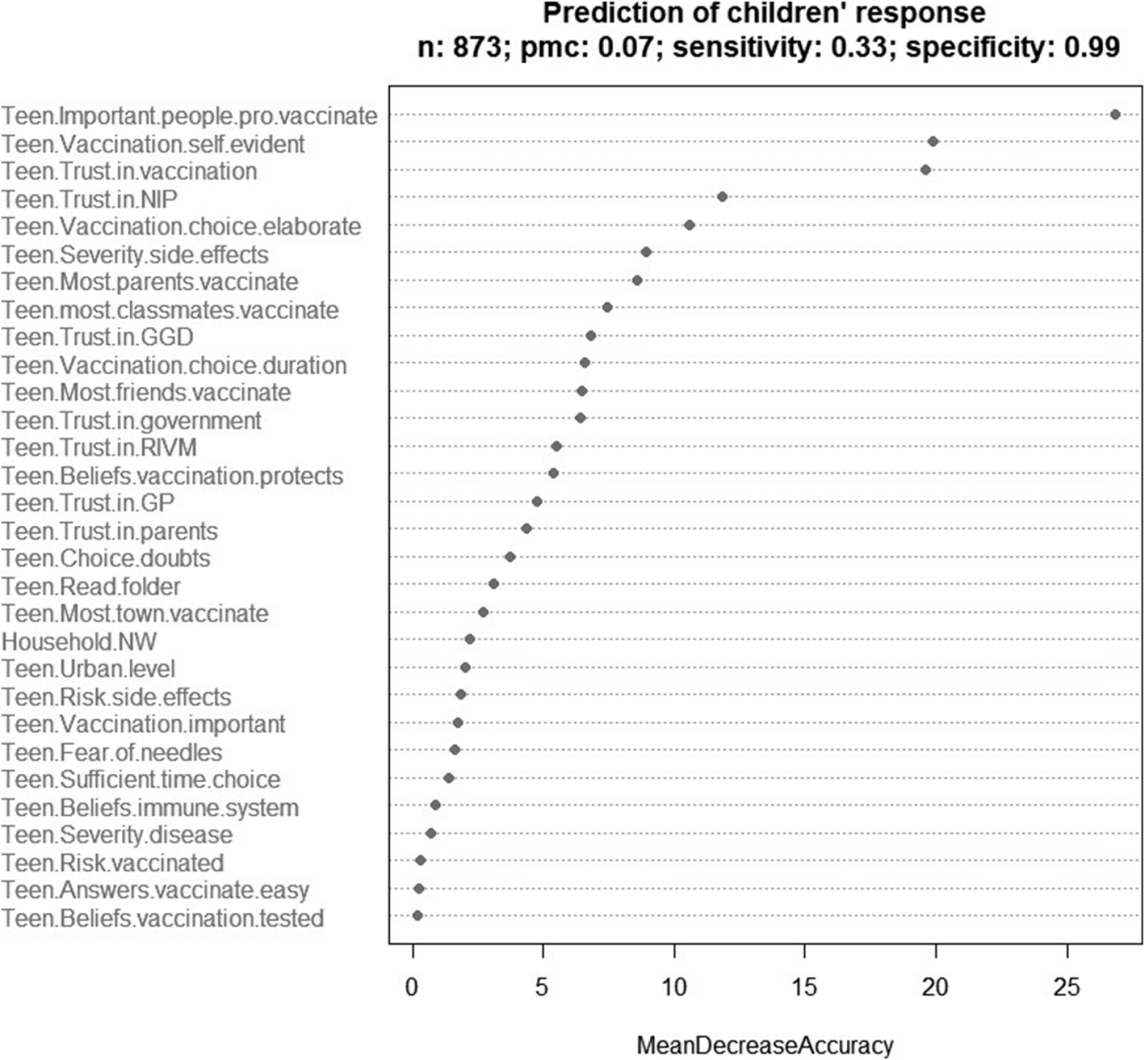
Prediction analysis: variable importance for MenACWY vaccination uptake among adolescents s (*N* = 873). The Mean Decrease in Accuracy of a given predictor variable is the decrease in the proportion of correct predictions regarding MenACWY vaccination uptake that results from randomly permuting the values of that variable in the dataset

The RF model for adolescents has a sensitivity of 33%, a specificity of 99% and a pmc of 0.07. The results of the prediction analyses, including the ranking of the predictor variables according to their importance, are summarized in Figs. 3 and 4. Analyses based on random subsets of the data with more balanced proportions of vaccinated and unvaccinated show essentially the same variable importance and yield much better sensitivities at the cost of somewhat lower specificities. These sensitivities and specificities are closer to those obtained by trying to balance the two quantities in the corresponding ROC analyses. We note the large values of the area under the curve in the two analyses. Additional file A shows the ROC analyses and provides further information (see Additional file A).

Among parents several factors stand out, centring on the process of the decision (having doubts, the duration of making a choice, vaccinating as self-evident), their attitude about the MenACWY vaccination (the necessity and importance of the vaccination), trust in the vaccination and lastly ideas of important people around them (injunctive norm).

Among adolescents the three stand-out predictors are the ideas of important people around them (injunctive norm), the process of the decision (vaccinating as self-evident) and trust in the vaccination.

These predictive factors emphasise that there are differences found among those that have vaccinated and those that have not, and this goes for both the parents and the adolescents. In particular, the RF results show a high probability of correctly predicting influences that led to people getting vaccinated, while the sensitivity of both models underscore that they not fully capture factors that predict why people do not get vaccinated.

Parents of nonvaccinated adolescents had more doubts about making their decision, with simple logistic regression showing the odds of an adolescent not getting vaccinated to be higher among parents who experienced doubts about their choice compard to parents who did not experience doubts (OR = 4.87, *p* < 0.001, CI[95%] 3.35 – 7.03, *n* = 1,091).

### No-shows and reasons for refusing MenACWY vaccination

Those invited multiple times for getting MenACWY vaccination were asked about why they did not go on the first opportunity offered. A wide variety of reasons were mentioned why people had not gone on the first offered opportunity. Scheduling issues with the appointment was the most mentioned reason. Other reasons stated were wanting more time to delve into the topic, it being circumstantially too close to another vaccination, the adolescent’s fear of needles or long waiting times at their first attempt. This thus indicates practical barriers, information needs and beliefs can be at play. Of those that had received multiple invitations, half of their adolescents were vaccinated after all, demonstrating that no-shows in the first round are not necessarily unwilling to vaccinate.

**Fig. 5:**
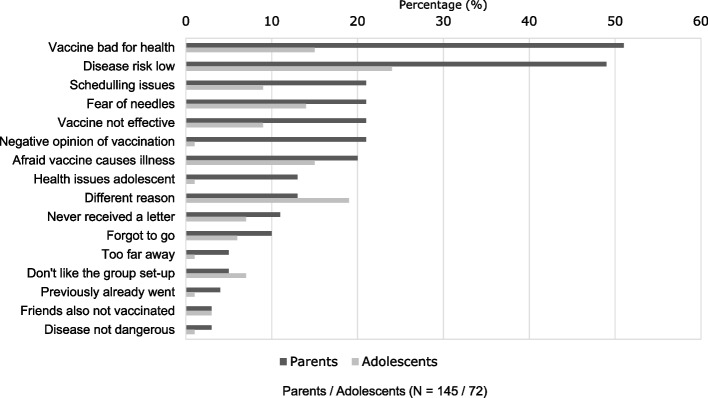
Reasons for not getting the MenACWY vaccination by parents (*N* = 145) and adolescents (*N* = 72)

Those that did not get vaccinated were asked about their reasons for not doing so with a multiple answer question. Figure 5 shows the reasons given by parents and adolescents for not getting the vaccination. Most reported answers among parents were that the vaccine was not good for the health of their child, and that the risk at getting the disease was low. The latter was also most reported by adolescents. What was striking was that solely parents mentioned the reason having generally a negative opinion about vaccinations and adolescents experiencing health issues that prevent vaccinating.

### Within the dyads: comparing parent and adolescent from the same household

We aimed to collect data from both the parent and the adolescent in each household to compare their answers and get an idea of where they might disagree on things. Table 2 in paragraph 3.1 displays demographic data on this subset of respondents from our study population.

This subset of data is used to assess if there were any intra-household differences by comparing the answers given by both in the dyad. Table 4 shows on which statements there was either most or least agreement and the direction of that disagreement. Adolescent scores were deducted from parental scores to calculate what is referred to as the dyad score. A dyad score of 0 means they gave the same answer, a positive score means the parent scored higher and a negative score means the adolescent scored higher.

**Table 4:** Percentages of dyad where parent and adolescent score similar, where parent scored higher than adolescent or adolescent scored higher than parent within the same household for various statements

Measure	Item	Answer option/range	% DYAD with similar score	% DYAD where parent scored higher	% DYAD where adolescent scored higher
			*score difference* = *-1/0/1*	*score difference* = *2 or higher*	*score difference* = *-2 or lower*
Engagement	I find vaccinating against meningococcal disease an important topic	1 = completely agree to 5 = completely disagree	72.14	1.58	26.28
Risk perception (not vaccinated)	Imagine that your son or daughter did not/you did not vaccinated against MenACWY. How big do you think the chance is that your son or daughter/you will get meningococcal disease?	1 = very small to 5 = very large	79.84	5.14	15.02
Risk perception (vaccinated)	Imagine that your son or daughter/you did get vaccinated against MenACWY. How big do you think the chance is that your son or daughter/you will get meningococcal disease?	1 = very small to 5 = very large	97.04	1.38	1.58
Severity of disease	How severe do you think meningococcal disease is?	1 = not severe to 5 = very severe	89.72	8.30	1.98
Side effects	How big do you think the chance is that someone experiences side effects after getting the MenACWY vaccination?	1 = very small to 5 = very big chance	81.82	4.55	13.64
	If there are side effects after the vaccination, how severe do you think these might be?	1 = not severe tot 5 very severe	77.87	7.51	14.62
Beliefs	The MenACWY vaccination offers good protection against menincgococcal disease	1 = completely agree to 5 = completely disagree	86.96	4.94	8.10
	A vaccination is only offered to people once its safety has been very thoroughly tested	1 = completely agree to 5 = completely disagree	84.39	6.92	8.70
	When the immune system of a child is good, they are less likely to get meningococcal disease	1 = completely agree to 5 = completely disagree	71.55	20.55	7.91
Attitude towards the MenACWY vaccination	The vaccination against meningococcal disease is…	1 = very bad to 5 = very good	92.89	5.73	1.38
		1 = very unimportant to 5 = very important	90.71	6.72	2.57
		1 = very unnecessary to 5 = very necessary	91.10	7.31	1.58
Deliberation regarding the choice for/against the MenACWY vaccination	Getting the vaccination against meningococcal disease for my son or daughter/myself is…	1 = very much self-evident to 5 = very much not self-evident	90.91	4.15	4.94
	Getting the vaccination against meningococcal disease for my son or daughter/myself is something I thought about a…	1 = very short amount of time to 5 = very long amount of time	82.51	10.93	6.56
	I thought about the decision whether to get my son or daughter/myself vaccinated against meningococcal disease…	1 = very briefly to 5 = very extensively	60.92	26.85	12.22
Doubts	During the decision making for the MenACWY vaccination, I have had…	1 = no doubts, 5 = a lot of doubts	91.30	4.15	4.55
Trust	MenACWY vaccine	1 = little trust to 5 = a lot of trust	92.69	5.53	1.78
	NIP	1 = little trust to 5 = a lot of trust	88.74	9.09	2.17
	RIVM	1 = little trust to 5 = a lot of trust	90.52	6.52	2.96
	Government	1 = little trust to 5 = a lot of trust	89.93	7.50	2.57
	GGD	1 = little trust to 5 = a lot of trust	88.34	9.09	2.57
	GP	1 = little trust to 5 = a lot of trust	89.53	4.74	5.73
	Online experiences of others	1 = little trust to 5 = a lot of trust	75.30	4.94	19.76
	Personal experiences of others	1 = little trust to 5 = a lot of trust	78.66	3.56	17.79

Within the dyads there is the most misalignment on perceived time spent thinking about the MenACWY decision (the parent deliberated longer), on the importance of the topic (the adolescent is less engaged), chance at getting the disease when not vaccinated (adolescents perceived higher chance), chance at side effects and that side effects were severe once vaccinated (adolescent perceived higher chance and severity), on the belief that a strong immune system can prevent meningococcal disease (the parent more strongly disagreed) and on the trust placed in personal experience stories online or in person (the adolescent reported more trust in these).

Instances of dyad agreement for most other factors are high, with the greatest alignment found in the perception of risk once vaccinated, the severity of the disease, beliefs that vaccination offers good protection or not and that vaccination is safe or not, in attitudes towards the MenACWY vaccination, in the self-evident aspect of getting vaccinated or not, having a lot of doubts or not, and having trust or not in the MenACWY-vaccination, NIP, RIVM, government, GGD, and GP.

Parents and adolescents did not always agree on who was involved in the final decision regarding the MenACWY vaccination in their household, with the biggest disagreements found concerning the adolescent’s role in the final decision.

Among the dyads, 49% of parents and 56% of adolescents claimed the final decision was made by both parents and the adolescent together. While nearly 30% of dyad parents claimed no adolescent involvement at all in the final decision, only 13% of dyad adolescents claimed the same. Among the dyads where the adolescent stated they, together with both parents, decided on the MenACWY vaccination (*n* = 281), just over a third (37%) of their parents disagreed and claimed there was actually no role for the adolescent in the final decision.

Where adolescents stated they themselves decided on the MenACWY vaccination alone (*n* = 89), only 20% of their parents agreed this was actually the case.

## Discussion

Through the retrospective look at people’s choice concerning the MenACWY vaccination, factors have been identified that best predict the outcome of MenACWY vaccination choices for both parents and adolescents during the catch-up campaign. Similar factors important in decision-making among both parents and adolescents were finding vaccination self-evident, ideas of important people around them that vaccinating is a good thing and having trust in the vaccination. Other important factors among parents were not having doubts, short duration of making a choice, and attitude towards MenACWY vaccination.

A study done in the Netherlands before the MenACWY catch-up campaign looked at the influence of knowledge and beliefs on intent to vaccinate against meningococcal disease among parents and adolescents [26]. They concluded that beliefs about vaccinations in general, more than specific beliefs about meningococcal disease and the MenACWY vaccination best predicted the intention to vaccinate against meningococcal disease among parents and adolescents. In addition, vaccination intention of the parents was also an important predictor for the intention to vaccinate among adolescents. In our study, by retrospectively looking at people’s choice, beliefs about vaccination seemed to be less important, instead factors such as attitude towards and trust in the MenACWY vaccination, and factors that occur during the decision-making process (e.g., doubts and deliberation) were important. What did prove congruent, also with other studies among adolescents in the Netherlands, was parental influence and the importance of the parent’s position in the adolescent’s reasoning [26, 43].

The dyad data showed, amongst others, that parents compared to adolescents spent more time thinking about the MenACWY decision and were more engaged with the topic. This is perhaps not surprising as adolescents experience decision-making in ways that might differ from adults or might be more prone to focus on short-term effects [40, 41].

The greatest alignment was found in the perception of risk getting meningococcal disease once vaccinated, in attitudes towards the MenACWY vaccination, in the self-evident aspect of getting vaccinated or not and in trust in the MenACWY-vaccine, NIP, RIVM, government, GGD en GP. The latter two factors were also influencing the choices about MenACWY vaccination among parents and adolescents.

Combining the RF analyses with findings from the dyad data shows that for the influential factors that best predict choices made concerning MenACWY, dyads are mostly in agreement, except for that adolescents spend less time thinking about making a choice than parents. In fact, this factor is only influential among parents in their decision-making process in such that parents who deliberate less about MenACWY vaccination more often decide to vaccinate their child.

The dyad data demonstrates that while the decision-making within households is often done by both parents and the adolescent together, the latter’s influence and participation is sometimes viewed differently within a dyad. There are instances where the adolescent claimed participation in the final decision or even sole say in the matter, while the parent stated this was not the case. Thus whether adolescents find the topic important or not, or if they play a role in the decision-making or not, might not actually alter much in terms of the final decision. This proved to be in line with previous research [42, 43].

Comparisons made within the dyads also show the mother played a bigger role in the decision-making process than the father, with the mother being the parent mostly spoken to before a decision is made. Fathers’ involvement in child vaccination decisions, and specifically how this might affect vaccination uptake has not been widely explored [44–46]. This dynamic could also benefit from further research to explore why this is the case and what this might mean for household level decisions concerning adolescent vaccinations.

### Practical implications

Our study showed that parents have prominent influence in the decision-making about whether their child is getting the MenACWY vaccination or not, while the adolescent’s influence in the household decision-making is more limited. Therefore, information about MenACWY vaccination might be mainly addressed to the parents of the teenagers and whereby the dialogue about MenACWY vaccination between parents and teenagers will be stimulated.

Despite using similar information sources, parents of vaccinated and nonvaccinated adolescents placed different levels of trust in the folder sent along with the invitation to get vaccinated, the RIVM website and the news. Although perhaps trust in these sources is not easily improved, it is valuable to be aware of the potential impact of a source being viewed as less trustful as it might influence how people interpret the information provided [47].

Furthermore, conversations with a GP or the provider of the vaccination (GGD/JGZ) make highly rated contributions to choices made among parents who make use of these. However, these options are not widely used among our study population. Other research has shown the importance of contact with health care providers to positively affect uptake among parents with doubts about vaccination [48]. Raising the frequency of use of certain sources, especially those deemed very reliable among households, might prove a useful strategy to solidify vaccination uptake numbers. Especially as those who choose not to get the MenACWY vaccination reported experiencing more doubts throughout their decision-making.

Simultaneously, social media was not often used to gain information, either by parents or by adolescents. And those that did use it, rated it as not very trustworthy. The dyad data showed that adolescents reported to have more trust in personal experience stories online or in persons compared to parents. This might be different in the underrepresented groups in our study and future research on social media influence on adolescent vaccination decision-making could lay bare potentially different dynamics.

Not everyone got the vaccination at the first opportunity offered to them, with a number of households indicating having received more than one invitation. Difficulty with scheduling was the most mentioned reason for missing the first appointment opportunity. As half of the households who had received multiple invites got the vaccination at a later time, it is important to remember that sometimes things that have nothing to do with how people think about vaccinations, and that sending reminders may be very important for increasing vaccine uptake.

### Strengths and limitations

Including parents and adolescents from the same households allowed us to reflect on the differing perspectives about the decision-making process within a household and to look at this from both the parent’s and the adolescent’s perspective.

Furthermore, we gathered data after people had gone through their decision-making process concerning the MenACWY vaccination. Intent is an important predicator for future behaviour, but the retrospective approach used in this study allowed respondents to assess not what they think might be important to them, but to ascertain what in fact played a role in the decision made. This offers insight into how respondents went about making the actual decision and get a clearer sense of what mattered in their decision-making. However, this does open up the possibility of recall bias as people might misremember how things transpired.

Another limitation concerns our initial goal to include 500 parent-adolescent dyads who had chosen to get the MenACWY vaccination and 500 parent-adolescent dyads who had chosen not to get it. Despite our efforts to oversample respondents who had not received the MenACWY vaccination, persons with a lower SES and persons with a migration background, the majority of respondents for this study got the vaccination, was higher educated and from Dutch origin. In addition, the majority of respondents were from high urbanised areas as we included all eligible postal codes from three large cities in the Netherlands. We therefore cannot generalise our findings to the Dutch population of parents and adolescents. Furthermore, these sampling and response biases may have influenced somewhat the order and/or strength of the factors influencing people’s choice about MenACWY vaccination, but less on which factors were important, which was our main interest.

Lastly, one of the reasons for the low response rate among certain groups might be the use of online surveys in that certain population groups either choose not to participate or do not have the appropriate conditions to participate. This prevented us from making comparisons in the decision-making processes based on these demographics. It would be worthwhile to perform offline studies in specific groups to find out whether similar factors are important for vaccination decision-making and then also specific approaches and methods could be used.

## Conclusion

Parents have prominent influence in the decision-making, while the adolescent’s influence in the household decision-making is more limited and often less than the adolescents themselves perceive it to be. Furthermore, adolescents tend to be less engaged and spend less time thinking about the decision compared to parents. Therefore, information about MenACWY vaccination might be mainly addressed to the parents of the adolescents and whereby the dialogue about MenACWY vaccination between parents and adolescents will be stimulated.

Predictive factors for getting the MenACWY vaccination among both adolescents and parents were finding vaccination self-evident, ideas of important people around them about that vaccinating is a good thing, and having trust in the vaccination. With regard to the factor trust, raising the frequency of use of certain sources, especially those deemed very reliable among households such as conversations with a GP or the provider of the vaccination (GGD/JGZ), might prove a useful strategy to solidify vaccination uptake numbers.

Opinions of parents and adolescents from the same households concerning the factors that are influential to them, do not differ a lot in the final decision-making for the MenACWY vaccination.

## Supplementary Information

**Additional file 1.**

## Data Availability

The data that support the findings of this study are stored in the data repository of The National Institute of Public Health and the Environment (RIVM). The data are not publicly available. Data are however available from the authors upon reasonable request and with permission of the National Institute of Public Health and the Environment (RIVM).

## Abbreviations

IMD: Invasive meningococcal disease
MenW: Meningococcal disease serogroup W
MenACWY: Meningococcal disease strains A, C, W and Y
NIP: National Immunisation Programme
ROC: Receiver-Operator Characteristics
HPV: Human papillomavirus\
RIVM: National Institute for Public Health and the Environment in the Netherlands
METC: Medical Ethics Review Committee
SES: Socioeconomic status
PAP: Precaution Adoption Process
RF: Random forest
OR: Odds Ratio
GP: General Practitioner
GGD: Municipal Health Services
JGZ: Youth Health Services
