# Supplementary material for: Adolescent and parental decision-making for the MenACWY vaccination: influential predictors and parental-adolescent differences among households in the Netherlands
Source: BMC Public Health. 2023 May 25;23:947. doi: 10.1186/s12889-023-15872-9 (PMC10210315; doi:10.1186/s12889-023-15872-9)
Supplement: Supplementary file 1 — Additional file 1. [file 12889_2023_15872_MOESM1_ESM.docx]

Figure A - Receiver operating characteristics (ROCs) of the prediction of the vaccination status of adolescents based on data of parents by random forests (RF).


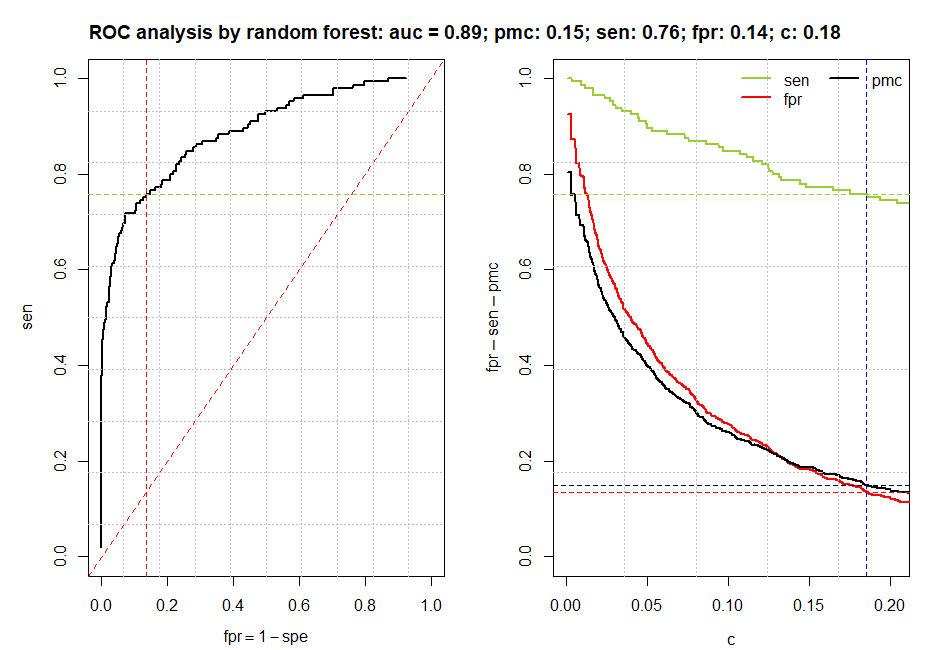


Figure B - Receiver operating characteristics (ROCs) of the prediction of the vaccination status of adolescents based on data of adolescents by random forests (RF).


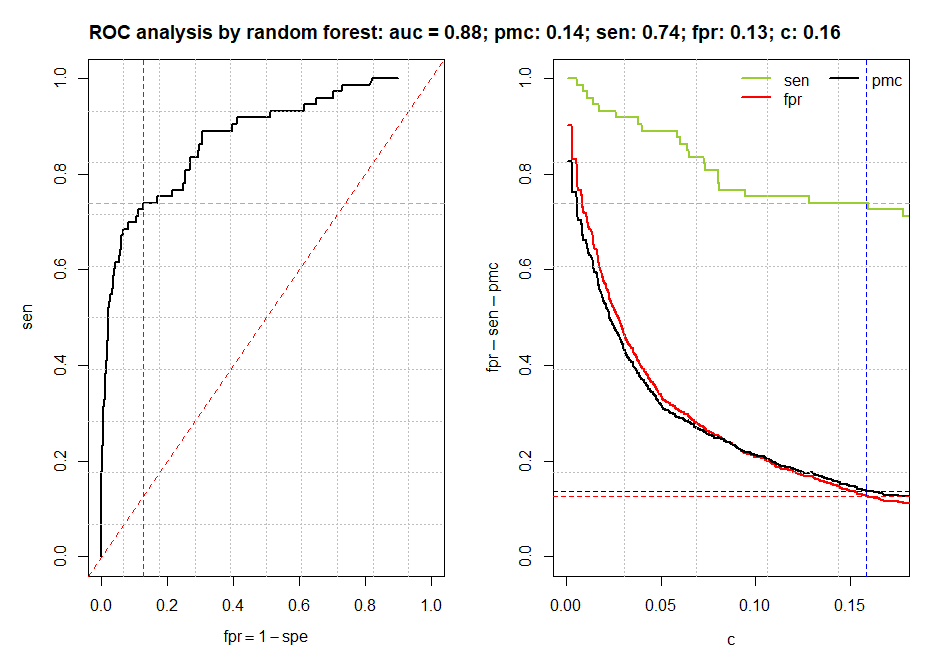


Additional file notes

The RF algorithm estimates an individual’s ratio of the probability of not getting vaccinated to the probability of getting vaccinated conditionally on the individual’s (or on the parent’s) predictor variables and then predicts the individual’s vaccination status as 1 (not vaccinated) if that ratio is $\geq1$ and as 0 (vaccinated) otherwise. This rule may be changed by predicting the individual’s status as 1 if and only if the estimated ratio is $\geq c$, where $c$, the ‘cutoff point’, is allowed to take values other than 1. By varying the values of $c$, the RF yields different prediction rules with varying values of the performance indicators. The combined values of two of these indicators, namely the sensitivity (sen) and the false positive rate (fpr, equal to 1 minus the specificity, or spe), constitute the ROC curve, shown on the left panels; the values of pmc (probability of misclassification), sen and fpr are shown as functions of $c$ on the right panels. A rule that may be thought to be convenient is the one that balances the sensitivity and specificity in such a way as to have them the farthest northwest possible along the ROC curve, albeit at the cost of a somewhat higher value of pmc. The cut-off point yielding that particular rule is indicated as the dashed vertical line on the right panels; in both cases its value lies between 0.15 and 0.2, reflecting the need for the RF to promote the prediction of individuals into the non-vaccinated group (albeit with due regard to their predictor variables) as a means of compensating for the small size of the latter. The large values of the auc (area under the ROC curve) and the other performance indicators witness the substantial value of an individual’s data to predict their vaccination status, and they support the results concerning the stronger predictor variables.
